# A first record of a suspected intestinal myiasis caused by *Muscina stabulans* (Fallén 1817) (Diptera: Muscidae) in Southern South America

**DOI:** 10.1017/S0031182024001094

**Published:** 2024-09

**Authors:** Fernando H. Aballay, Marta I. Saloña-Bordas, M. Alejandra Perotti

**Affiliations:** 1 Departamento de Biología, Facultad de Ciencias Exactas y Naturales, Instituto y Museo de Ciencias Naturales, Universidad Nacional de San Juan, San Juan, Argentina; 2 Consejo Nacional de Investigaciones Científicas y Técnicas, San Juan, Argentina; 3 Universidad del País Vasco / Euskal Herriko Unibertsitatea, UPV/EHU, Apdo 644 Leioa, Bilbao, España; 4 Ecology and Evolutionary Biology Section, School of Biological Sciences, University of Reading, Reading, UK

**Keywords:** accidental myiasis, baby host, dipteran, maggots, muscinae, toddler

## Abstract

We report a case of a suspected intestinal myiasis of a child from Southeast Argentina. Diptera larvae were sampled by a physician from the nappy worn by the child and submitted for examination and identification to the Laboratorio de Artrópodos (Universidad Nacional de Mar del Plata). Based on diagnosis of the anterior and posterior spiracles and mouthparts, the larvae were identified as the false stable fly *Muscina stabulans* (Fallén, 1817) (Diptera: Muscidae). A comparison of diagnostic characters between Argentinean and European third instars of this species is presented. *Muscina stabulans* is a prevalent species in the district of General Pueyrredón, Buenos Aires province, where the case occurred. Its abundance in the area coincidentally peaked at the time of the infestation. This is the 1st report of *M. stabulans* as a suspect of intestinal myiasis for the whole of the southern cone of South America (Chile and Argentina).

## Graphical abstract

**Figure:**
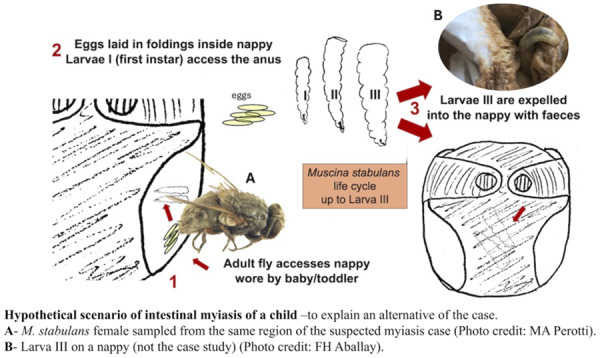

## Introduction

Myiasis is produced by some fly larvae (Diptera) feeding on live tissues of humans and other animals (Hope, 1840). According to James (1947), there are two major types of myiasis, which are defined by (i) the species of Diptera producing myiasis, or, by the (ii) the body parts/tissues affected. In the case of Muscidae and Fanniidae species (Diptera), myiasis is considered accidental and the targeted organ is the intestinal duct (enteric myiasis) (James, 1947). These larvae will feed on live tissues producing damage on the host with the risk of infections. Once third instar larvae have finished feeding, they migrate, abandoning the food source or live tissues for pupation. The damage to the living tissues increases and favours the reaction of the host and the detection and attempts of elimination of the larvae. Gastrointestinal myiasis is a type of myiasis where the fly larvae feed on the epithelium of the digestive tract. It can be produced by accidental ingestion of fly larvae, through contaminated food, but, more frequently by exposure or presence of feces and/or other human excretions that work as attractant to the myiasis female flies, which will lay their eggs or first instar maggots directly on the skin in proximity of or in the anal opening (Desoil and Delhaye, 1922; Causey, 1938; Kenney, 1945; James, 1947).

The false stable fly *Muscina stabulans* (Fallén, 1817) is a Diptera of the family Muscidae, subfamily Muscinae, whose larvae commonly occur in animal and human excrements as well as in decomposing corpses and carcasses. *Muscina stabulans* is also a facultative and opportunistic myiasis species colonising living animals (Skidmore, 1985; Hall and Smith, 1993). This species is associated with intestinal myiasis, and the type of myiasis is considered a pseudo-myiasis (Aspöck, 1972; Hall and Smith, 1993), or, accidental myiasis by the early literature (James, 1947).

Larvae of *M. stabulans* are also cannibalistic and prey on other fly larvae as *Musca domestica* L. do, but mainly on fly species that share same or similar habitats and food resources (Skidmore, 1985; Hall and Smith, 1993; Pinto Duarte *et al*., 2013). Worldwide, *M. stabulans* is a cosmopolitan housefly (also known as a filth fly and as the false stable fly) associated with animal rearing facilities in temperate regions. *M. stabulans* occurs in the Americas breeding in manure or dung of poultry, livestock (beef or dairy cattle) and swine farms (Axtell, 1986; Perotti, 1998).

In this work, we were able to analyse larvae from a suspected case of human intestinal myiasis, which host was a ca. 1 year old child. The myiasis arthropod species was identified and circumstances that might favour this type of infestations in Southest East Argentina were assessed.

## Materials and methods

### Description of the case

A family vacationing in a suburban resort area of the District of Gral. Pueyrredón, Buenos Aires province, Argentina, admitted their distressed, ca.1-year old child to the local paediatrics hospital, ‘HIEMI Materno Infantil Don Victorio Tetamanti’ of Mar del Plata. The vacation took place during the spring, in November of 1998. The paediatrician on call, who examined the child, made the following observations which were passed onto the entomologists who studied the invertebrate: 1. A case of diarrhoea with the presence of an unidentified intestinal parasite within the nappy; 2. During defecation, the unidentified parasites were defecated as small batches of large live fly larvae together with the feces; 3. The child suffered of diarrhoea for the last day before been taken to the hospital; 4. Information on the location of the family vacation and the times. No further information was provided about the duration of time the child was wearing a nappy. However, there were no reports of parental neglect, and the parents took the child for examination immediately after realizing the ‘illness’, suggesting the nappies were changed timely.

### Examination of samples

Three of the collected larvae by the paediatrician (at the hospital) were preserved in pure alcohol (100% ethanol) and sent to us, to the Laboratorio de Artrópodos, Facultad de Ciencias Naturales, Universidad Nacional de Mar del Plata -which is located in the specific region (Southeast Argentina, Buenos Aires province), for species identification. The 3 larvae were received by the senior author, MAP, while she was developing a project on filth flies at the Universidad Nacional de Mar del Plata (in 1998).

### Identification of species

To confirm the identification, samples of the same suspected species, *M. stabulans,* of a laboratory colony at the nearby Universidad de Quilmes were provided for comparison. The colony at Quilmes was originated from captures of flies within the same region. Therefore, larvae specimens of the Quilmes lab colony were studied and compared to the preserved larvae collected from the case by the paediatrician.

The 3 preserved maggots from the myiasis case and approx. 40 larvae from the colony (all local/same region populations) were observed under a stereo microscope at different magnifications. Larvae were cleared following Wolff Echeverri (2006): soaked in 10% KOH/Water v/v for 24 h; then in 5% Glacial Acetic Acid/Water v/v for 13 min; followed by 2 washes of distilled water for 30 min; 30% Ethanol/Water v/v for 30 min, and finally fixed in 70% Ethanol/Water v/v. Larvae were dissected ventrally for detailed examination of external and internal morphologies. The dissected morphologies, cephalopharyngeal skeleton and spiracles were located on microscope slides for observations and photographs (Stereo: Leica s9D, camera: Leica DM 2700 P). Specialised keys were used for the identification of the dipteran larvae, particularly based on distal structures and cephalopharyngeal skeleton (Skidmore, 1985; de Queiroz and de Carvalho, 1987; Grzywacz *et al*., 2017). In addition to the identification of the larvae, adults emerging from reared larvae from the colony were thoroughly, further identified as *M. stabulans* using the works of Nihei and Dominguez (2008) and Patitucci *et al*. (2013).

## Results

**Figure 1.**
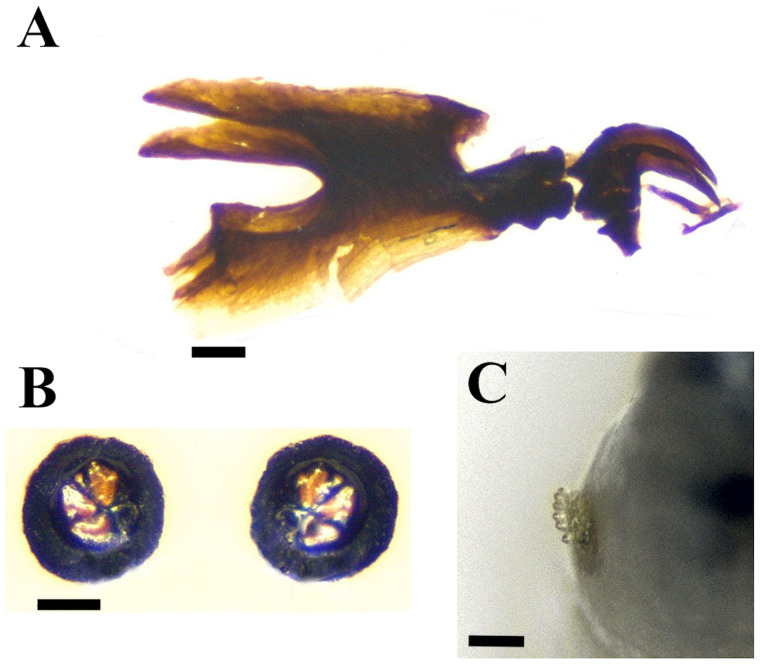
*Muscina stabulans* (Buenos Aires-Argentina populations), dissected morphologies: (A) Cephaloskeleton; (B) Posterior spiracles, mature larva; (C) anterior spiracle. Size bar A–C: 0.1 mm. (Photos' credit: Fernando H. Aballay).

After examination of the 3 larvae from the case plus larvae from the laboratory colony, all these were identified as third instar larvae of *M. stabulans* (Diptera: Muscidae: Muscinae) (Fig. 1). For these larvae, morphological variations, when compared to those of other populations were observed in the sclerotization of the posterior spiracles and mouthparts (Fig. 1). These Argentinean larvae of *M. stabulans* showed clear differences from the material studied by Grzywacz *et al*. (2015), from European populations, as follows:

− the larvae in the studied material from Buenos Aires-Argentina populations have thick and shorter buccal teeth (Fig. 1A) in comparison with illustration 2E, *M. stabulans,* in Grzywacz *et al*. (2015).
− the dorsal spine in the intermediate sclerite, in the Argentinean larvae it is thinner and towards the front, with little sclerotization. While Grzywacz *et al*. (2015) illustrations 1D and 2E show a rounded spine and central to the intermediate sclerite;
− in postfeeding larvae, posterior spiracles are well sclerotised (Fig. 1B), with thick peritreme, quite similar to those of illustration 2F (Grzywacz *et al*., 2015). While analysing third instar feeding larvae, the posterior spiracles are less sclerotised, with thinner peritreme of radiating slits marked, which could be confused with larvae III of *M. prolapsa* in illustration 2D. The morphological differences between these examined larvae with other *M. stabulans* populations from Europe (Grzywacz *et al*., 2015, 2017) might respond to adaptations to the local environment and can be seen as population variations. As mentioned in methods, a parallel confirmation of the species, adults emerging from the same reared larvae batches (lab colony) were confirmed as *M. stabulans* by the entomologists at Quilmes University (Dr N. Centeno and M. Chirino, see acknowledgements).

Another observation made by the entomologist assessing the preserved larvae from the myiasis case, was the presence of blood-red contents inside the larvae midgut canal of the 3 larvae, as seeing by transparency throughout the cuticle -via stereo microscopy, pre-dissection, at the time of arrival in the lab. This finding made us suspect that these larvae might have been feeding off the child's intestinal epithelium (living tissues) prior defecation (or prior the release by diarrhoea expulsion) to the nappy where they were collected together with the feces by the physician. The 3 larvae seemed to show signs of entering pupation. However, due to the lack of information it cannot be confirmed that they were post feeding larvae. They could equally be feeding Larvae III, prematurely expulsed in the diarrhoea events. In any case, the visual assessment of the internal contents of the midgut was clear and showed bright red contents.

## Discussion

Perianal or within-nappy oviposition of gravid *M. stabulans* is suspected. In this potential scenario, female gravid flies might have accessed the inside of the child nappy, or perhaps ovipositing on little openings or crevices. Therefore, some of the hatched first instar larvae that might have moved towards the anus, invaded through the terminal end of the enteric channel or rectum. Once there, larvae are capable of feeding on living tissues too, likely epithelium, continuing developing. They either stop feeding and migrate or are forcefully released with feces independently of their age. Although the type myiasis cannot be confirmed by the entomologists -who only analysed the insects, myiasis cannot be completely ruled out based on the comments of the paediatrician, who suggested a case of intestinal myiasis (intestinal ‘parasite’). We still consider relevant reporting this case as it would be the first for southern South America, establishing precedence for further similar situations. An alternative explanation would consider that gravid flies managed to lay eggs inside the nappy, then, larvae developed up to their 3rd instar feeding off the feces. However, the process of development to reach larvae III requires more than 1 day, up-to several days. This scenario is not feasible for the discussed case considering that the nappies were changed very regularly, every day. In this case scenario only 1st instar larvae and eggs, if at all, would have been found together with the feces, never 3rd instars.

Only a handful of cases of intestinal human myiasis caused by the fly *M. stabulans* have been documented and reported from around the world. Interestingly, a number of those cases involved small children: Bernhard (1987) and Benecke and Lessig (2001) from Germany; Soliman *et al*. (2003) from the UK; Scott (1964) from USA; Mazzotti (1967) from Mexico, Pérez-Inigo (1974) from Brazil. In addition, two unconfirmed cases from India, Shivekar *et al*. (2008) and Udgaonkar *et al*. (2012) -as the identification of the maggots is contested (Bernhardt *et al*., 2019).

Early experimental studies about the development of intestinal and rectal myiasis in animals (Desoil and Delhaye, 1922; Causey, 1938) and in humans (Kenney, 1945) have shown that ingested maggots cannot survive the gastric acids at the beginning of digestion, inside the digestive tract. Pseudo-myiasis, is the term adopted in the case of accidental ingestion of immatures that consequently ‘would develop’ myiasis in the intestinal tract (Hall and Smith, 1993).

*Muscina stabulans* is also responsible of urethral myiasis (James, 1947; Aspöck, 1972), a phenomenon that can only occur by ovipositing around the urethral opening and not by ingestion. Intestinal and urethral myiasis are facultative myiasis. These are an occasional and temporary phenomenon that can last a few days until the 3rd instar maggots moult and leave the anus or urethra to pupate; or, could be ejected from the anus by diarrhoea, and even earlier. Treatment of enteric myiasis do not require specific medication. We suggest that enteric myiasis infestations in little children or toddlers would likely occur when gravid flies can access undisturbed and oviposit in the openings of nappies; eventually laying batches of eggs, enabling the first instar larvae to reach the anus. The warm body temperature promotes eggs to hatch in a very short time. First instar larvae could immediately start migrating and might find access to the anus, where they can feed and complete the first and second instar in a very short time, also favoured by the warm environment provided by the body. Some of the larvae will likely be feeding on the intestine epithelium, the living tissue. As this occurs, lesions and changes in the intestinal flora will produce inflammation and discomfort, and consequently diarrhoea. The fly life cycle will be completed when the third instar larvae move away from the feeding area seeking a dryer environment to pupate, or, larvae are forcefully expulsed as a reaction of the host in the feces during diarrhoea episodes. Any of these two scenarios could be proposed for the present case, with the third instar larvae released out of the intestinal tract together with feces.

The suburb resort where the family vacationed is located in proximity to one of the poultry farming areas in Argentina. The myiasis incident occurred in late November, towards the beginning summer -midway spring, which coincidentally overlaps with the peaks of abundance of *M. stabulans* in the region, being the most prevalent species of synanthropic muscid during the spring (Perotti, 1998; Labud *et al*., 2003).

This is the first report of *M. stabulans* associated with a suspected case of intestinal myiasis in Argentina, and for the whole of southern South America.

## Data Availability

All data is presented in the main manuscript. Materials such as voucher specimens of larvae III are kept at the Laboratorio de Entomología Aplicada y Forense, Universidad Nacional de Quilmes, Argentina.
